# Maturation of Monocyte-Derived DCs Leads to Increased Cellular Stiffness, Higher Membrane Fluidity, and Changed Lipid Composition

**DOI:** 10.3389/fimmu.2020.590121

**Published:** 2020-11-27

**Authors:** Jennifer J. Lühr, Nils Alex, Lukas Amon, Martin Kräter, Markéta Kubánková, Erdinc Sezgin, Christian H. K. Lehmann, Lukas Heger, Gordon F. Heidkamp, Ana-Sunčana Smith, Vasily Zaburdaev, Rainer A. Böckmann, Ilya Levental, Michael L. Dustin, Christian Eggeling, Jochen Guck, Diana Dudziak

**Affiliations:** ^1^Laboratory of Dendritic Cell Biology, Department of Dermatology, Friedrich-Alexander University Erlangen-Nürnberg (FAU), University Hospital Erlangen, Erlangen, Germany; ^2^Nano-Optics, Max-Planck Institute for the Science of Light, Erlangen, Germany; ^3^Max-Planck-Zentrum für Physik und Medizin, Erlangen, Germany; ^4^Department of Physics, Friedrich-Alexander University Erlangen-Nürnberg (FAU), Erlangen, Germany; ^5^Biological Optomechanics, Max-Planck Institute for the Science of Light, Erlangen, Germany; ^6^Science for Life Laboratory, Department of Women’s and Children’s Health, Karolinska Institutet, Stockholm, Sweden; ^7^MRC Human Immunology Unit, Weatherall Institute of Molecular Medicine, John Raddcliffe Hospital, University of Oxford, Oxford, United Kingdom; ^8^Roche Innovation Center Munich, Roche Pharmaceutical Research and Early Development, pRED, Munich, Germany; ^9^PULS Group, Department of Physics, IZNF, Friedrich-Alexander University Erlangen-Nürnberg (FAU), Erlangen, Germany; ^10^Mathematics in Life Sciences, Department of Biology, Friedrich-Alexander University Erlangen-Nürnberg (FAU), Erlangen, Germany; ^11^Medical Immunology Campus Erlangen, Erlangen, Germany; ^12^Computational Biology, Department of Biology, Friedrich-Alexander University Erlangen-Nürnberg (FAU), Erlangen, Germany; ^13^McGovern Medical School, The University of Texas Health Science Center, Houston, TX, United States; ^14^Kennedy Institute of Rheumatology, University of Oxford, Oxford, United Kingdom; ^15^Institute for Applied Optics and Biophysics, Friedrich-Schiller University Jena, Jena, Germany; ^16^Leibniz Institute of Photonic Technologies e.V., Jena, Germany; ^17^Deutsches Zentrum Immuntherapie (DZI), Erlangen, Germany; ^18^Comprehensive Cancer Center Erlangen-European Metropolitan Area of Nuremberg (CCC ER-EMN), Erlangen, Germany

**Keywords:** cell mechanics, cellular stiffness, lipids, lipidomics, monocyte-derived dendritic cells, maturation, low-density lipoprotein, cholesterol

## Abstract

Dendritic cells (DCs) are professional antigen-presenting cells of the immune system. Upon sensing pathogenic material in their environment, DCs start to mature, which includes cellular processes, such as antigen uptake, processing and presentation, as well as upregulation of costimulatory molecules and cytokine secretion. During maturation, DCs detach from peripheral tissues, migrate to the nearest lymph node, and find their way into the correct position in the net of the lymph node microenvironment to meet and interact with the respective T cells. We hypothesize that the maturation of DCs is well prepared and optimized leading to processes that alter various cellular characteristics from mechanics and metabolism to membrane properties. Here, we investigated the mechanical properties of monocyte-derived dendritic cells (moDCs) using real-time deformability cytometry to measure cytoskeletal changes and found that mature moDCs were stiffer compared to immature moDCs. These cellular changes likely play an important role in the processes of cell migration and T cell activation. As lipids constitute the building blocks of the plasma membrane, which, during maturation, need to adapt to the environment for migration and DC-T cell interaction, we performed an unbiased high-throughput lipidomics screening to identify the lipidome of moDCs. These analyses revealed that the overall lipid composition was significantly changed during moDC maturation, even implying an increase of storage lipids and differences of the relative abundance of membrane lipids upon maturation. Further, metadata analyses demonstrated that lipid changes were associated with the serum low-density lipoprotein (LDL) and cholesterol levels in the blood of the donors. Finally, using lipid packing imaging we found that the membrane of mature moDCs revealed a higher fluidity compared to immature moDCs. This comprehensive and quantitative characterization of maturation associated changes in moDCs sets the stage for improving their use in clinical application.

## Introduction

Dendritic cells (DCs) are very important professional antigen-presenting cells of the immune system (1). They are distributed throughout the body including lymphohematopoietic tissues such as the lymph nodes, thymus, tonsils, or spleen, but can also be found in barrier tissues such as the skin, brain, or mucosa (2, 3). DCs act as sentinels and form a bridge between innate and adaptive immunity (4). Upon recognition of pathogenic antigens, DCs upregulate costimulatory molecules and chemokine receptors, secrete cytokines, and migrate into secondary lymphoid organs (5–7). During these maturation steps, DCs process the acquired antigens and present small peptides in the major histocompatibility complex (MHC; in humans: human leucocyte antigen HLA) binding groove. T cells that recognize these peptide:MHC complexes in an inflammatory context — possibly correlating to the peptide-dependent flexibility of the peptide:MHC complex (8) — become activated and undergo proliferative responses, finally leading to immunity. In dependency of the DC subset that is presenting the antigen as well as the cytokine milieu, naïve T cells differentiate into different sets of T effector cells (9–12). Besides, DCs play also a pivotal role in regulating peripheral tolerance in the steady-state, as they are able to present self-antigens in a non-inflammatory environment and thus activate regulatory CD4^+^ T cells and induce a non-responsive state in T effector cells (1, 13, 14). Due to their exceptional ability to orchestrate T cell responses, DCs represent ideal targets for immunotherapeutic approaches (1, 2, 15–17).

In general, DCs are distinguished into conventional DCs type 1 (cDC1), type 2 (cDC2), and plasmacytoid DCs (3, 16, 18–20). While these populations are present under both, homeostatic and inflammatory conditions, the development of a cell population from monocytes sharing key phenotypic and functional characteristics with cDCs has been described exclusively in an inflammatory milieu *in vivo* (21). This population is regularly referred to as monocyte-derived DCs (moDCs), inflammatory DCs, or Tip DCs in the literature (16, 22, 23). In contrast to cDCs, monocytes exhibit a high frequency in human blood rendering them an interesting tool for the generation of vast moDC numbers for autologous cell transfer approaches (23–25). Further, the combination of robustness, functional parallels to primary DCs, and availability emphasize the application of moDCs as a model system to gain a general overview in new aspects of DC biology (26, 27).

The *in vitro* differentiation of moDCs from monocytes in the presence of granulocyte-macrophage colony-stimulating factor (GM-CSF) and interleukin-4 (IL-4) was established over two decades ago and allows for the generation of large amounts of moDCs (24, 28, 29). Following cultivation, moDCs can be matured *via* various stimuli including the application of agonistic αCD40 antibodies, TLR ligands (including LPS, (TLR4 ligand), poly (I:C) (TLR3 ligand), CpG (TLR9 ligand)), or cytokine cocktails (such as the classical maturation cocktail comprising IL-1β, PGE_2_, IL-6, and TNFα) (29, 30). Mature moDCs share a multitude of features with primary DCs in peripheral blood including a typical stellate morphology, high expression of MHC I and MHC II, and the ability of antigen presentation.

The principle of cell-based therapy using moDCs for self-vaccination against incurable tumors was established several years ago (23). *Ex vivo* loading of *in vitro* generated moDCs from patients using antigenic peptides, soluble proteins, tumor lysates, or RNA/DNA in combination with adjuvants to establish fully mature moDCs was applied to treat different solid tumor entities in otherwise incurable patients (2, 15, 31–35). Even though moDC-based therapies increased the life expectancy of certain types of cancer patients, the response rate is still lower than desired (15, 36–39). For DC vaccination, it is crucial that injected moDCs migrate into draining lymph nodes to meet their corresponding T lymphocytes. Several studies in melanoma patients revealed that after intradermal injection, only 2–4% of the moDCs reached the lymph nodes (40, 41). After intravenous injection in mice, it has been found that bone marrow-derived DCs were accumulating in vascularized organs such as spleen, lung, kidneys, and liver instead of migrating into lymph nodes (42, 43). This could be caused by virtue of inappropriate mechanical and cellular properties that might affect the microcirculation (44). Even after intranodal injections in melanoma patients, moDCs were found mostly nonviable and also less mature when accumulating in the lymph nodes (41, 45). This was reflected by insufficient immune responses and poor prognosis for the cancer patients (39). Thus, it will be important to better understand cell migration capacities and the circulation of cells in the lymphatic system and in blood for improving of moDC-based immunotherapies and induced T cell responses.

Cell migration is a key component for the function of DCs. In peripheral tissues, patrolling primary DCs sample their environment for antigens (immature DCs) and upregulate the C-C chemokine receptor 7 (CCR7) upon envisaging danger signals. Depending on the further activation status, migration speed might increase, enabling the DCs to travel into the draining lymph nodes to activate T cells (5, 46–48). Migration is an active process relying on actin and microtubule cytoskeleton remodeling that is accompanied with mechanical cellular changes (49). Pioneer work to address dynamics of the cytoskeleton during DC migration demonstrated that migration is dependent on non-muscular myosinIIA contractility generating the force for movement of bone marrow-derived DCs in confined microenvironments such as microfabricated channels (50–52). Further, active remodeling of the actin cytoskeleton and its regulatory proteins is a highly dynamic process including the maturation of DCs, antigen uptake by plasma membrane vesicle internalization to endo- and lysosomal compartments, antigen processing and presentation, recycling of MHC molecules, and DC-T cell interactions at the immunological synapse (48, 53–56). Various methods for analyses are available to measure mechanical properties of a cell such as atomic force microscopy, magnetic or optical forces, particle tracking microrheology, or traction force microscopy (57, 58). In contrast to the listed methods, the recently developed microfluidic lab-on-chip system, so-called real-time deformability cytometry, allows for high-throughput measurements of single cells in suspension to understand the processes linking cell mechanics with cell function (59).

All these steps also involve active membrane remodeling. Biomembranes provide a multitude of functions for cells as they establish a biological barrier to the extracellular environment and thus control the movement of molecules into and out of cells. The best-studied cellular membrane is the plasma membrane that consists of a lipid bilayer, which is tightly packed with various transmembrane proteins (60, 61). The distribution of different lipid species allows for the formation of functional microdomains, so-called lipid rafts (62–64). Distinct combinations of lipids, such as sterols and sphingolipids, with membrane proteins are essential in the processes of endocytosis, signal transduction, and many other membrane functions (62–65). The majority of mammalian plasma membranes are formed by glycerophospholipids, glycolipids, and sterols. i) The first group consists of amphipathic molecules, which share a common glycerol backbone and contain a phosphate group (66). This polar head group can be modified leading to the formation of different phospholipids: phosphatidylcholine (PC), phosphatidylethanolamine (PE), phosphatidylserine (PS), phosphatidylinositol (PI), and phosphatidic acid (PA). ii) Next, the major forms of sphingolipids are composed of sphingomyelin (SM) and glycosphingolipids (66). iii) Sterols constitute the third most abundant group of membrane lipids. One representative is cholesterol that is enriched in the plasma membrane where it interacts with sphingolipids. Therefore, cholesterol is able to modify the fluidity and structure of the membrane (67). Interestingly, asymmetrical distribution of lipids between the two lipid monolayers has been described to facilitate signaling into the cell and regulation of cellular processes (66). However, the multitude of different lipids and their complex function in cell homeostasis are poorly understood. Recently, a new high-throughput and sensitive mass spectrometry method has been introduced to quantitatively characterize the lipid composition of cells (lipidome) by lipidomics analysis (68, 69).

Since the lipid composition and therefore the formation of functional membrane microdomains and cytoskeletal changes facilitating DC migration might orchestrate the execution of a variety of DC tasks, we hypothesized that the maturation of DCs induces changes in the cytoskeleton as well as in the membrane properties. DCs might need some robustness to withstand mechanical forces including shear stress during circulation and migration into secondary lymphoid organs and while maintaining flexibility for cognate T cell interaction. Here, we show, to our knowledge for the first time, that mature moDCs exhibited a higher stiffness than immature moDCs due to changes in the cytoskeleton as measured by real-time deformability cytometry analyses. In order to gain more knowledge on global lipid changes in moDCs during maturation, as these are linked to membrane changes, we performed an unbiased high-throughput lipidomics screening of multiple donors. Our data demonstrate that moDCs undergo a great conversion of the overall lipid composition. Further, the identified lipid changes were associated with the serum lipid levels of moDC donors indicating an important role of LDL and cholesterol for DCs and the resulting immune responses. By investigating the plasma membrane using lipid packing imaging, we found that mature moDCs increased their membrane fluidity. Thus, we propose that the DC maturation process has an impact on the stiffness of moDCs and on membrane properties, both potentially contributing to efficient emigration and settlement of DCs in new tissue environments such as lymph nodes or tumors and mediating the consequent interaction with T cells.

## Materials and Methods

### Generation of Monocyte-Derived Dendritic Cells

Leucocyte reduction cones from ten healthy donors were obtained from the Department of Transfusion Medicine and Haemostaseology in Erlangen. From all donors, donor-matched serum was collected (see 2.3). Samples were received under local ethical committee approvals (Ethikkommission der Friedrich-Alexander-Universität Erlangen-Nürnberg), and informed written consents were obtained in accordance with the Declaration of Helsinki.

Monocyte-derived DCs (moDCs) were generated as described before (70, 71). Briefly, the blood of anonymous and healthy donors was diluted with RPMI1640 (Sigma). Peripheral blood mononuclear cells (PBMCs) were isolated by density centrifugation using Human Pancoll (ρ = 1.077 g/ml; PanBiotech). After centrifugation, the interphase containing PBMCs was collected and washed twice with RPMI1640. 5 × 10^7^ cells were adhered to γ-Globulin (50 ng/ml, Sigma) coated tissue culture dishes (TC-treated cell culture dish, 100 mm; BD Falcon) and incubated for 1 h at 37°C, 5% CO_2_, and 96% rH. The medium, containing the non-adherent fraction, was removed and 10 ml DC-medium (RPMI1640 containing 100 U/ml penicillin, 100 µg/ml streptomycin, 2 mM L-glutamine, 10 mM HEPES, and 2% human sera type AB (Lonza)) was added to the cells and incubated overnight at 37°C, 5% CO_2_, and 96% rH. On the next day, the medium was replaced with DC-medium containing 800 U/ml GM-CSF (Peprotech) and 50 U/ml IL-4 (Peprotech). On day 3 and day 5, a total of 4 ml fresh DC-medium supplemented with 800 U/ml GM-CSF and 50 U/ml IL-4 or 400 U/ml GM-CSF and 25 U/ml IL-4 was added, respectively. To analyze immature as well as mature moDCs, a maturation cocktail consisting of 13.2 ng/ml IL-1β (Peprotech), 1,000 U/ml IL-6 (Peprotech), 10 ng/ml TNFα (Peprotech), and 1 μg/ml PGE_2_ (Sigma) was added to one half of the cells for 24 h on day 6. On day 7, immature as well as mature cells were harvested. As fully differentiated moDCs are detaching from the culture dish when using the above described maturation protocol, the use of EDTA or cell scrapers is not needed. Thus, for mature moDCs the supernatant, which is containing mature cells, was harvested. Immature moDCs also detach from the bottom of the culture dish. However, they are more adhesive, requiring careful rinsing of the dish with the above layered medium. Afterwards, moDCs were stained for FACS analysis, cell sorting, or real-time deformability cytometry measurements.

### FACS Analysis and Cell Sorting of moDCs

On day 7, immature and mature moDCs were harvested and 5 × 10^5^ or 1 × 10^6^ cells were stained for FACS analysis or cell sorting, respectively. For FACS analysis, CD11c-PE/Cy7 (3.9, BioLegend), HLA-DR-BV605 (L243, BioLegend), CD83-A647 (HB15e, BioLegend), DEC205-PE (MG38, BD Bioscience), and DC-SIGN-FITC (CD209, DCN46, BD Bioscience) antibodies in PBS/2% human sera or the respective isotype controls (IgG1-A647 (MOPC-21, BioLegend), mouse IgG2b-PE (MPC-11, BioLegend), and mouse IgG2b-FITC (eBM2b, eBioscience)) were applied to the cells for 15 min on ice in the dark. After washing, the cells were resuspended in PBS/2% human sera + 0.1 µg/ml 4′,6-diamidino-2-phenylindole and acquired using a 5-laser line BD LSRFortessa.

For cell sorting, moDCs were harvested and stained with an antibody cocktail consisting of the following antibodies in PBS/2% human sera for 20 min on ice: HLA-DR-BV510 (L243, BioLegend), CD83-PE (HB15e, BioLegend), CD86-FITC (2331(FUN-1), BD Biosciences), CD11b-PE/Cy5 (M1/70, BioLegend), CD11c-PE/Cy7 (3.9, BioLegend), CD14-Alexa700 (HCD14, BioLegend). After washing, cells were resuspended in PBS/2% human sera + 0.1 µg/ml 4′,6-diamidino-2-phenylindole and cell sorted using a BD FACSAria II cell sorter into immature (CD83^-^CD86^low^) and mature (CD83^+^CD86^high^) moDCs. 4.5 × 10^5^ cells of each population were sorted, washed with PBS and 3,000 cells/µl were cryopreserved at −80°C until they were sent to Lipotype for lipidomics analysis.

### Serum Samples

In addition to leucocyte reduction cones, each 7.5 ml blood of ten donors was obtained in serum tubes from the Department of Transfusion Medicine and Haemostaseology in Erlangen. After coagulation, the blood was centrifuged at 3,000xg for 10 min without brake and the serum was used for analyzing lipid content. Cholesterol, low-density lipid-cholesterol (LDL-cholesterol), high-density lipid-cholesterol (HDL-cholesterol), triglycerides, lipoprotein a, and lipase were measured in the Department of Clinical Chemistry in the Central Laboratory in Erlangen, the serum was stored at 4°C until measurement. Free fatty acids and beta-hydroxybutyrate were analyzed in the Clinical Laboratory of the Paediatric Clinic in Erlangen. Therefore, serum samples were kept on ice until measurement since free fatty acids are not stable.

### Cell Mechanics Measurements Using Real-Time Fluorescence and Deformability Cytometry

Real-time fluorescence and deformability cytometry measurements were performed on moDCs as described previously (59, 72). Briefly, immature and mature moDCs were harvested on day 7 of culture. First, 1 × 10^6^ cells were stained with the antibodies CD83-PE and HLA-DR-FITC for 10 min at RT to ensure that only immature (CD83^low^) or mature (CD83^high^) cells were taken into account during the measurement. After washing moDCs with PBS/2% human sera, the supernatant was aspirated, and cells were resuspended in PBS supplemented with 0.5% (w/v) methylcellulose (Sigma Aldrich) to increase medium viscosity. The sample was flushed through a microfluidic channel constriction 20 µm x 20 µm in cross section by applying a constant flow rate of 0.16 µl/sec. An image of every measured cell was taken by a high-speed camera and beside other parameters, cell deformability and projected area (cell size) were calculated. Data analysis was performed using the software ShapeOut (Zellmechanik Dresden) and gating for an area ratio between 1 and 1.05 as well as an area gate between 90 to 600 µm² were used to exclude wrongly detected events and debris, respectively. Furthermore, moDCs were gated into immature (CD83^low^) and mature (CD83^high^) cell populations. Statistical analyses were carried out using 1D linear mixed model that incorporates fixed effect parameters and random effects to analyze differences between cell subsets and replicate variances, respectively. p-values were determined by a likelihood ratio test, comparing the full model with a model lacking the fixed effect term.

### Lipidomics Analyses

#### Lipid Extraction for Mass Spectrometry Lipidomics

Mass spectrometry-based lipid analysis was performed by Lipotype GmbH (Dresden, Germany) as described (73). Lipids were extracted from 1.2 × 10^5^ to 2.3 × 10^5^ sorted immature and mature moDCs from 10 donors using a two-step chloroform/methanol procedure (69). Samples were spiked with internal lipid standard mixture containing: cardiolipin 16:1/15:0/15:0/15:0 (CL), ceramide 18:1;2/17:0 (Cer), diacylglycerol 17:0/17:0 (DAG), hexosylceramide 18:1;2/12:0 (HexCer), lyso-phosphatidate 17:0 (LPA), lyso-phosphatidylcholine 12:0 (LPC), lyso-phosphatidylethanolamine 17:1 (LPE), lyso-phosphatidylglycerol 17:1 (LPG), lyso-phosphatidylinositol 17:1 (LPI), lyso-phosphatidylserine 17:1 (LPS), phosphatidate 17:0/17:0 (PA), phosphatidylcholine 17:0/17:0 (PC), phosphatidylethanolamine 17:0/17:0 (PE), phosphatidylglycerol 17:0/17:0 (PG), phosphatidylinositol 16:0/16:0 (PI), phosphatidylserine 17:0/17:0 (PS), cholesterol ester 20:0 (CE), sphingomyelin 18:1;2/12:0;0 (SM), and triacylglycerol 17:0/17:0/17:0 (TAG). After extraction, the organic phase was transferred to an infusion plate and dried in a speed vacuum concentrator. The first-step dry extract was resuspended in 7.5 mM ammonium acetate in chloroform/methanol/propanol (1:2:4, V:V:V) and the second-step dry extract in 33% ethanol solution of methylamine in chloroform/methanol (0.003:5:1; V:V:V). All liquid handling steps were performed using the Hamilton Robotics STARlet robotic platform with the Anti Droplet Control feature for the pipetting of organic solvents.

#### Mass Spectrometry Data Acquisition in Lipidomics Analysis

Samples were analyzed by direct infusion on a QExactive mass spectrometer (Thermo Scientific) equipped with a TriVersa NanoMate ion source (Advion Biosciences). Samples were analyzed in both, positive and negative ion modes with a resolution of R_m/z = 200_ = 280,000 for mass spectrometry and R_m/z = 200_ = 17,500 for tandem mass spectrometry experiments, in a single acquisition. Tandem mass spectrometry was triggered by an inclusion list encompassing corresponding mass spectrometry mass ranges scanned in 1 Da increments (74). Both, mass spectrometry and tandem mass spectrometry data were combined to monitor cholesterol ester (CE), diacylglycerol (DAG), and triacylglycerol (TAG) ions as ammonium adducts; phosphatidylcholine (PC), phosphatidylcholine ether (PC O-), as acetate adducts; and cardiolipin (CL), phosphatidate (PA), phosphatidylethanolamine (PE), phosphatidylethanolamine ether (PE O-), phosphatidylglycerol (PG), phosphatidylinositol (PI), and phosphatidylserine (PS) as deprotonated anions. Mass spectrometry only was used to monitor lyso-phosphatidate (LPA), lyso-phosphatidylethanomlamine (LPE), lyso-phosphatidylethanolamine ether (LPE O-), lyso-phosphatidylinositol (LPI), and lyso-phosphatidylserine (LPS) as deprotonated anions; ceramide (Cer), hexoslylceramide (HexCer), sphingomyelin (SM), lyso-phosphatidylcholine (LPC), and lyso-phosphatidylcholine-ether (LPC O-) as acetate adducts.

#### Analyses of Lipidomics Data

Data were analyzed by Lipotype with in-house developed lipid identification software based on LipidXplorer (75, 76). Data post-processing and normalization were performed by Lipotype using an in-house developed data management system. Only lipid identifications with a signal-to-noise ratio >5, and a signal intensity 5-fold higher than in corresponding blank samples were considered for further data analysis. Extended raw data of lipidomics analysis is provided in the **Supplementary Material**. Exploratory analysis of lipidomics data was performed and visualized using R software (version 4.0.1, ×64, Linux, library gplots). Clusters in the standardized data were identified by means of two-way hierarchical clustering, where distances between data points were measured using Pearson’s correlation coefficient ρ as d=(1-ρ)/2 and the linkage criterion was chosen as minimal average distance between clusters. Principal component analysis (PCA) was used for feature extraction and dimensionality reduction.

### Lipid Packing Imaging

In order to perform lipid packing imaging, immature and mature moDCs (day 7) were spiked with a final concentration of 0.4 μM with Di-4-ANEPPDHQ (ThermoFisher) and subsequently incubated for 5 min on ice (77). In the next step, cells were washed with PBS. RPMI1640 without phenol red and serum was applied to the cells and the solution was transferred into 8-well chamber slides for imaging. The spectral imaging was performed using a Zeiss LSM780 confocal microscope equipped with a 40×, 1.2NA objective, and a 32-channel GaAsP detector array. Fluorescence excitation of Di-4-ANEPPDHQ was set to 488 nm and the lambda detection range was set between 500 and 700 nm. The values of the 32 channels were analyzed within the ImageJ plug-in “Stacks-T functions-Intensity vs. Time Monitor”. To calculate generalized polarization (GP) values, one has to define the wavelengths *λ*_Ld_ and *λ*_Lo_ of maximum emission of a probe in a reference liquid‐disordered (Ld) and liquid‐ordered (Lo) membrane environment. The wavelengths λ = 650 nm as maximum wavelength in disordered membraned (red shifted) and λ = 560 nm as maximum wavelength in the gel phase (blue shifted) were used as described previously (77–79).

$$GP=\frac{I_{560}-I_{650}}{I_{560}+I_{650}}$$

### Statistics

Statistical significances were either calculated within paired t-test, or ANOVA using GraphPad Prism 5. Not significant (n.s.) p>0.05, *p<0.05, **p ≤ 0.01, ***p ≤ 0.001.

## Results

### moDC Maturation Changes Mechanical Properties

**Figure 1:**
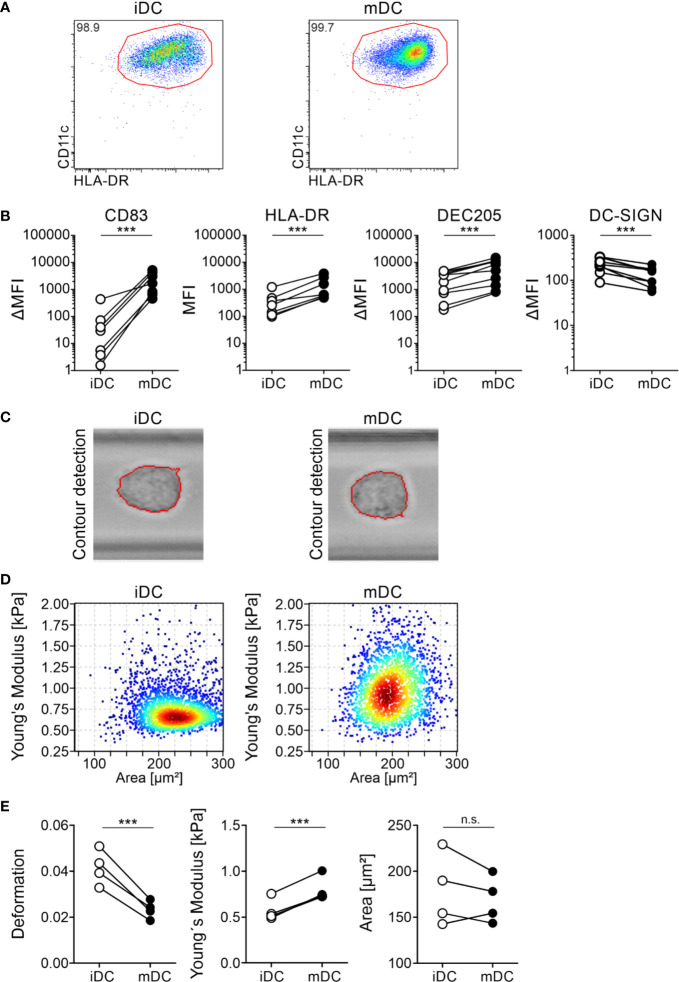
Differential protein surface expression and cell mechanics of immature and mature moDCs. moDCs were generated from monocytes of healthy donors within seven days in cell culture in the presence of GM-CSF and IL-4. On day 6 half of the cells were activated using a maturation cocktail consisting of PGE_2_, TNFα, IL-1β, and IL-6. On day 7 immature (iDC) and mature (mDC) moDCs were harvested and 5 × 10^5^ cells were stained for FACS analysis with the antibodies CD11c-PE/Cy7 (1:100), HLA-DR-BV605 (1:100), CD83-A647 (1:100), and CD86-PE (1:100), or respective isotype controls. Cells were recorded using BD LSRFortessa and analyzed using FlowJo software. **(A)** Gating of double positive CD11c^+^HLA-DR^+^ immature and mature moDCs. Depicted is the gating of one representative donor. **(B)** Paired scatter plots of expression of activation markers CD83 and HLA-DR on immature and mature moDCs as well as the DC markers DEC205 and DC-SIGN. Median fluorescence intensity ΔMFI results of the MFI of antibodies against CLRs minus MFI of the corresponding isotype controls. Statistical significances were calculated using paired t-test (n≥10). Not significant (n.s.) p>0.05, *p<0.05, **p ≤ 0.01, ***p ≤ 0.001. **(C–E)** Real-time fluorescence deformability cytometry of immature (iDC) and mature (mDC) moDCs. moDCs were generated as described before. On day 7, immature and mature moDCs were harvested and 1 × 10^6^ cells were stained for real-time fluorescence deformability cytometry analysis with the antibodies CD83-PE (1:100) and HLA-DR-FITC (1:50). Real-time fluorescence deformability cytometry of immature and mature moDCs from one representative donor (n = 4). **(C)** Phase-contrast images of one representative immature and mature cell with contour detection showing deformation of moDCs. **(D)** Young’s modulus with (elastic modulus E) of immature and mature moDCs of the same representative donor as in **(C)**. **(E)** Paired scatter plots of deformation, Young’s modulus and cell size (area) of all donors. Statistical significances were calculated using linear mixed model (n = 4). Not significant (n.s.) p>0.05, *p<0.05, **p ≤ 0.01, ***p ≤ 0.001.

A variety of cellular changes occur during the maturation of moDCs, such as upregulation of costimulatory molecules, CCR7-dependent migration, or a decrease of phagocytosis. We hypothesized that DCs need to change their cell mechanics when they mature as they emigrate from the peripheral tissue to move into the nearest lymph node, where they present the uptaken and processed antigens to T cells. These mechanical changes are dependent on cytoskeleton modifications (80). Therefore, to first investigate the mechanical properties of DCs, we focused on the model system of monocyte-derived DCs, which we generated from plastic-enriched peripheral blood monocytes cultured with GM-CSF and IL-4. On day 6, we incubated the cells with a maturation cocktail containing IL-1β, PGE_2_, IL-6, and TNFα (mature moDCs) or left the cells untreated (immature moDCs). moDCs were stained for DC-specific surface markers to perform flow cytometry as well as cell sorting. Our flow cytometry data ensured the generation of double positive CD11c^+^HLA-DR^+^ moDCs (**Figure 1A**) and demonstrated the upregulation of the surface markers HLA-DR, DEC205 (Ly75, CD205), and CD83 as well as down regulation of the marker DC-specific ICAM3-grabbing non-integrin (DC-SIGN, CD209) in mature moDCs (**Figure 1B**). Thus, our protocols allowed for moDCs differentiation and maturation.

To investigate the cell mechanical properties of immature and mature moDCs, we employed real-time fluorescence deformability cytometry measurements. This is a high-throughput method for the mechanical characterization of single cells in which a laminar flow in a 300 µm long microfluidic channel applies hydrodynamic forces to cells. Thus, immature and mature moDCs were generated and directly applied without further DC enrichment. As high speed imaging and processing allows for real-time recording of deformation and size (projected area) (59), contour detection of moDCs in the 300 µm long channel at the region of interest revealed that the cells got deformed on a microsecond timescale (**Figure 1C**). Furthermore, real-time deformability cytometry was found to be highly sensitive to cytoskeletal changes (80). Depending on the cell size different forces are exerted. To analyze the retrieved data, a full numerical model was applied that decouples size and deformation to quantitatively relate cell deformation to mechanical parameters (81, 82). Therefore, we calculated for all donors the Young’s modulus (elastic modulus E), which is inversely correlating with the degree of deformation, as a measure for cellular stiffness. Our data revealed that immature moDCs had a mean Young’s modulus of 0.573 ± 0.154 kPa, whereas mature moDCs were stiffer with a modulus of 0.799 ± 0.260 kPa (**Figures 1D, E**). Moreover, we also found a large variation in cell size in between the donors in our experiment. However, there was a slight trend towards larger immature moDCs. Taken together, our results suggest that mature moDCs exhibit a higher cell cortex stiffness than immature moDCs and that the overall cell mechanics changed during maturation.

### Shotgun Lipidomics Approach Identifies Lipid Composition Changes During moDC Maturation

**Figure 2:**
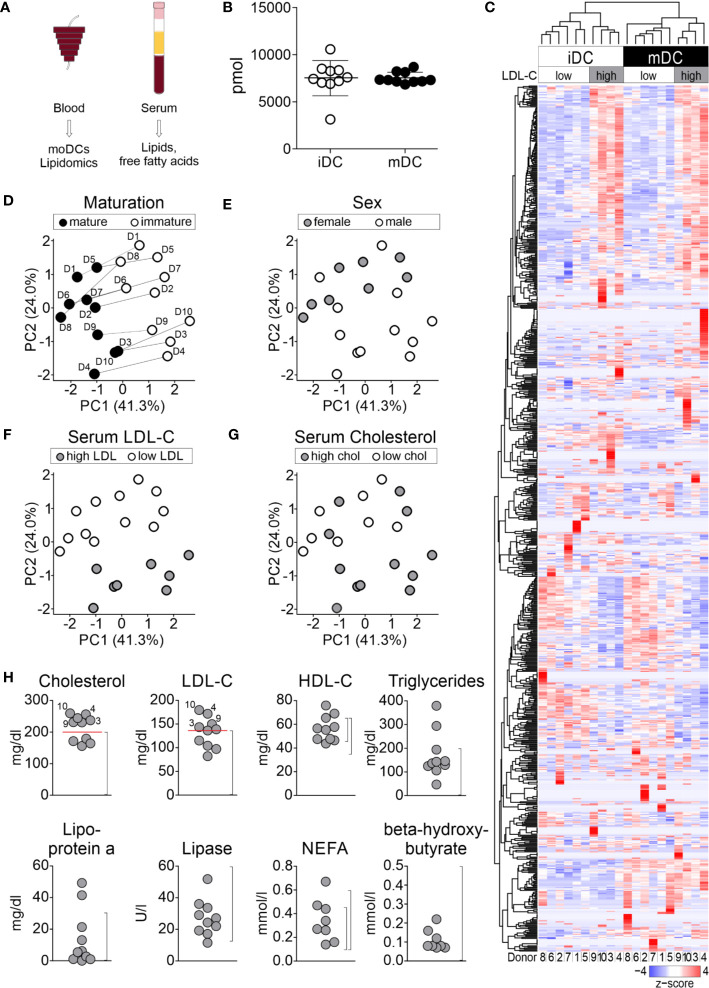
Lipid analyses of moDCs. moDCs were generated as described before. Immature and mature moDCs of ten healthy blood donors were sorted by flow cytometry and shotgun lipidomics analysis of 1.2 × 10^5^ to 2.3 × 10^5^ cells using mass spectrometry was performed by Lipotype Dresden. **(A)** Workflow for lipid analyses. **(B)** Global lipid content of immature and mature moDCs of all donors. The total amount of lipids was calculated by summing the pmol values of the individual lipids belonging to each donor. Values represent the mean of ten biological replicates. **(C)** 830 different lipids were detected, which make up the dendrogram on the left hand side of graph. Each column represents one sample: white indicates immature moDCs, while black depicts mature moDCs. The lipid amounts of cells have been normalized and scaled to a minimum of −4 and maximum of 4. Hierarchical clustering was performed using average linkage, where the distance between clusters has been calculated using Pearson’s correlation coefficient ρ (d=(1-ρ)/2). Principal component analysis (PCA) for identification of **(D)** maturation, **(E)** sex, **(F)** serum LDL-C, and **(G)** serum cholesterol-dependent lipid composition of moDCs. Lipid species mol% values per sample were used as input data and further analyzed using R software. Shown are the two principal components (PCs) that had the highest contribution to the variance within the data set. PC1: 41.3%, PC2: 24.0%. **(H)** Analysis of lipids and fatty acids for determination of overall serum lipid profile. Dot plots of serum lipid levels from blood donors for cholesterol (normal rage (NR): <200 mg/dl), low-density lipoprotein (LDL-C, NR: <140 mg/dl), high-density lipoprotein (HDL-C, NR female: 45–65 mg/dl, NR male: 35–65 mg/dl), triglycerides (NR: <200 mg/dl), lipoprotein a (NR: 0–30 mg/dl), lipase (NR: 13–60 U/L), non-esterified amino acids (NEFA, NR female: 0.10–0.45 mmol/L, NR male: 0.10–0.60 mmol/L), and β-hydroxybutyrate (NR: <0.5 mmol/L). Normal ranges for each lipid or fatty acid are indicated in brackets. Analyzed donors n = 10 (same donors as for lipidomics).

Beside changes in the actin/myosin cytoskeleton compartment (50, 83, 84), we hypothesized that also alterations in the overall lipid contents might contribute to the maturation process of moDCs. To retrieve unbiased information about all lipid changes during moDC maturation, we performed a shotgun lipidomics analysis. Therefore, immature and mature moDCs of ten healthy blood donors were generated, FACS-sorted into HLA-DR^+^CD11c^+^ cells, and cryopreserved. In addition, serum samples of the same donors were collected at the time of leucocyte apheresis for further analysis of lipids in the blood (see below, **Figure 2A**). In total, 830 lipids were detected by shotgun lipidomics mass spectrometry. Data post-processing and normalization were performed by the company Lipotype using an in-house developed data management system. Only lipids with an intensity 5-fold above the noise in mass spectrum and 5-fold above the intensity in blank samples were included in the analysis. Raw data from lipidomics analysis is provided in the **Supplementary Material**. Our data revealed that the total amount of cellular lipids did not change significantly after moDC maturation as immature moDCs and mature moDCs exhibited either 7,498 ± 1,870 pmol or 7,385 ± 600 pmol of total lipids, respectively (**Figure 2B**).

Thus, we next asked whether immature and mature moDCs, while having similar total lipid content, exhibit differences in the composition from individual lipid species. Therefore, we performed a two-way hierarchical clustering analysis using R software. Using this method, the clusters are built without any bias from the bottom up, starting with individual samples, calculating their pairwise distance and forming a cluster of the two closest samples. This process of calculating distances and pairing the closest clusters is repeated, forming larger and larger clusters until eventually the two remaining clusters are linked. The hierarchy is represented as dendrogram indicating which clusters are linked at which level. For the cluster analysis of lipidomics data we applied the Pearson correlation coefficient ρ as distance measure (d=(1-ρ)/2). At each step, the two clusters with minimal average distance were linked (average linkage clustering). Having performed this analysis, the data displays a clear separation of immature moDCs from mature moDCs at the highest level, which translates as follows: The average correlation of lipid compositions between immature and mature moDCs is lower than the average correlations within both clusters. Looking beyond the top-level clustering, we made another interesting observation: Within both the immature moDC and mature moDC clusters, sub-clusters have been formed separating donors 1, 2, 5, 6, 7, and 8 from donors 3, 4, 9, and 10 (**Figure 2C**).

The split of donors into two groups hints at the existence of another variable besides maturation status that contributes to the lipid distribution. To further investigate this phenomenon, we next performed principal component analyses (PCA) to reduce the complexity of the data set. Thus, the number of degrees of freedom was narrowed down by linear combination of different characteristics into principal components (PCs). Focusing on the PCs with the largest eigenvalues (i.e. PC1 and PC2) allows for the visualization of the multi-dimensional data in a two-dimensional graph (**Figures 2D–G**). Integrating our metadata including sex, serum lipid content (see below), and maturation status of the moDCs, we found that the maturation status demonstrated the highest impact to explain the variance within the data sets (PC1, 41.3%, PC2, 24.0%) (**Figures 2D–G**). Furthermore, our data demonstrated that gender had no significant impact on the data structure.

To investigate which other components might be masked in the PCA of the lipidomics screen, we started correlation analyses applying clinical parameters of the donors. Therefore, we analyzed the serum of the donors, from whom the moDC cultures were originally generated. In total, eight parameters were assessed, including cholesterol, LDL-cholesterol (LDL-C), HDL-cholesterol (HDL-C), triglycerides, lipoprotein a, lipase, non-essential fatty acids (NEFA), and beta-hydroxybutyrate. Upon visualization of the normal range, we found that 4 out of 10 donors could be classified as high LDL-C donors (>140 mg/dl, donors 3, 4, 9, and 10) and 6 out of 10 into low LDL-C donors (<140 mg/dl, donors 1, 2, 5, 6, 7, and 8). Another group could be determined, in which 6 out of 10 donors displayed a high total cholesterol (further mentioned as cholesterol) content >200 mg/dl (donors 3, 4, 5, 7, 9, 10) and 4 out of 10 had a low total cholesterol content <200 mg/dl (donors 1, 2, 6, 8) (**Figure 2H**). When we now used these parameters and overlaid them in the PCA analysis (**Figures 2D–G**), we found that the lipid changes of differentiated moDCs were directly correlating with the originally obtained serum lipid levels of LDL-C and cholesterol (**Figures 2D, F**) in that high serum LDL-C donors clearly separated from the other blood donors, indicating that serum LDL-C levels preserved lipid characteristics, independently from the maturation status of moDCs.

Further, the correlation with total cholesterol levels in the sera revealed a similar impact on the overall lipid composition of cells for high LDL-C and high cholesterol donors (donors 3, 4, 9, and 10). Interestingly, donors 5 and 7 displayed high cholesterol levels, but low LDL-C serum levels, and were thus not clearly correlating in the PCA analysis. For all other measured serum lipid levels, values of lipids were fluctuating between the donors (**Figure 2H**). Taken together, our data indicate that not only the maturation itself determines the lipid composition of moDCs, but also the serum LDL-C level influences the lipid composition, even after 7 days of controlled cell culture.

### Lipid Class Composition Shifts After Maturation of moDCs

**Figure 3:**
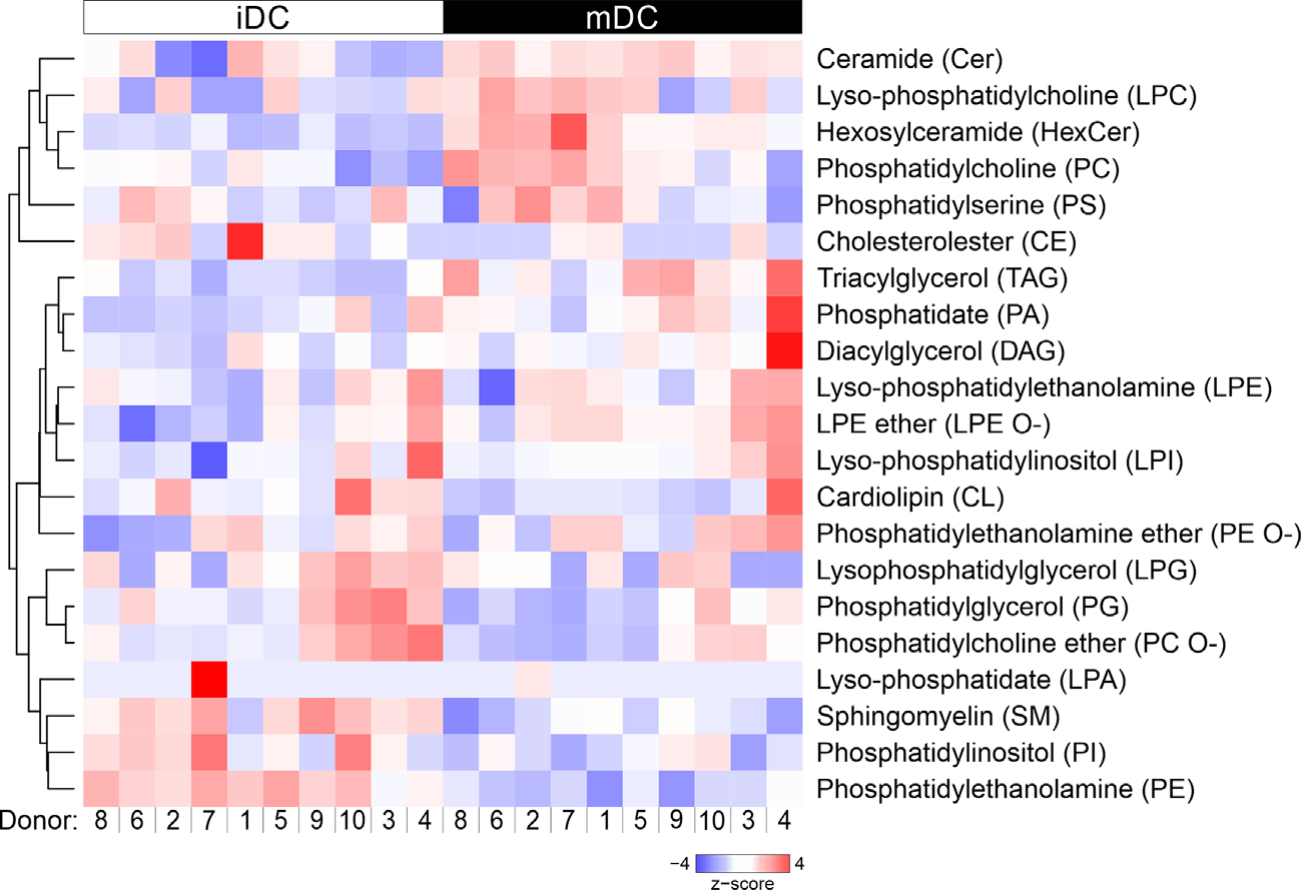
Hierarchical clustering of lipid classes. Mass spectrometric analysis of total lipid composition of immature and mature moDCs was conducted. Here, lipid species were grouped into 20 different lipid classes, which resulted in the dendrogram on the left hand side. Each column represents one sample: white indicates immature moDCs, whereas black depicts mature moDCs. The order of donors follows the clustering from **Figure 2C**. The lipid amounts of cells have been normalized and scaled to a minimum of −4 and maximum of 4. Hierarchical clustering of lipid classes was performed using average linkage, where the distance between clusters has been calculated using Pearson’s correlation coefficient ρ (d=(1-ρ)/2).

**Figure 4:**
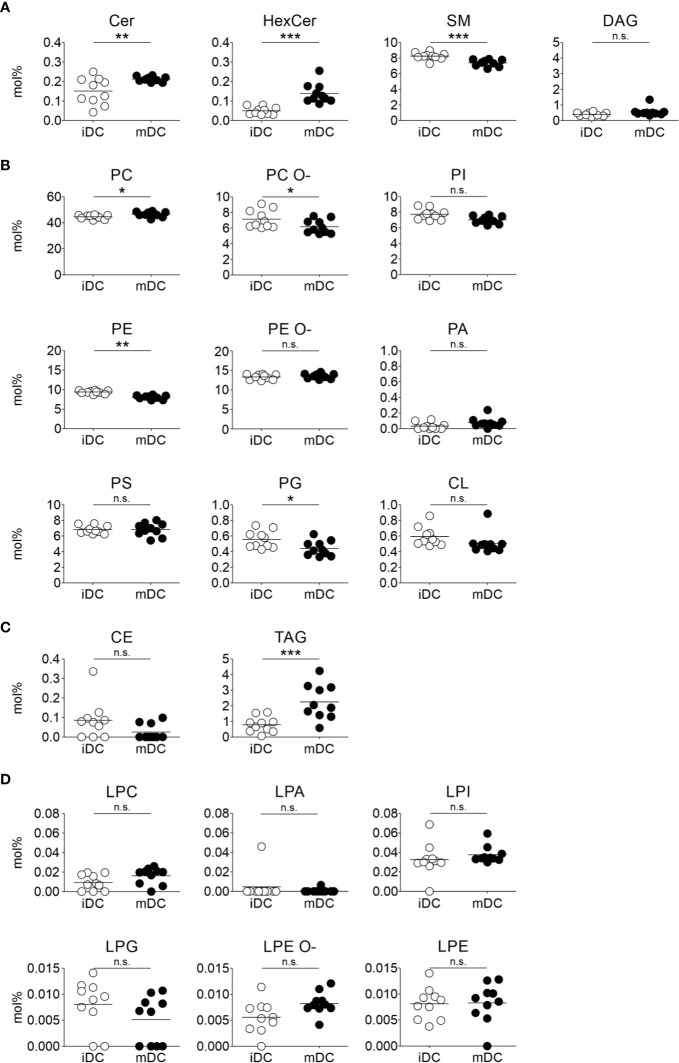
Lipid class changes upon maturation of moDCs. Mass spectrometric analysis of total lipid composition of immature and mature moDCs was carried out. Scatter plots demonstrate lipid class amounts for immature and mature moDCs of all donors. The amount of a lipid class (mol%) is calculated by summing the pmol values of the individual lipids belonging to each class. The class amount was then normalized to total lipid content. Horizontal lines represent mean values of the biological replicates. Content of membrane lipids is divided in **(A)** sphingolipids and glycerolipids, and **(B)** glycerophospholipids. **(C)** Lipid content of storage lipids and **(D)** messenger lipids in immature and mature moDCs. Statistical significances were calculated using ANOVA (n≥10). Not significant (n.s.) p > 0.05, *p < 0.05, **p ≤ 0.01, ***p ≤ 0.001.

We next assessed lipid class differences in moDCs after maturation. The quantified lipid species were grouped into storage lipids (triacylglycerol and cholesterol ester) and membrane lipids (sphingolipids, glycerophospholipids), which differ in their biological function and structure (66, 85, 86). Our hierarchical cluster analysis of combined lipids revealed that membrane lipids were significantly increased in ceramide (Cer) and hexosylceramide (HexCer), as well as in phosphatidylcholine (PC) in mature moDCs. In contrast, sphingomyelin (SM), phosphatidylcholine ether (PC O-), phosphatidylethanolamine (PE), and phosphatidylglycerol (PG) content decreased after moDC maturation (**Figures 3** and **4A, B**). Moreover, when we investigated the storage lipids in more detail, we found a statistically significant increase of triacylglycerol (TAG) in mature moDCs (**Figures 3** and **4C**). On the other hand, the messenger lipids lyso-phosphatidylcholine (LPC), lyso-phosphatidylethanolamine ether (LPE O-), lyso-phosphatidylethanolamine (LPE), lyso-phosphatidylinositol (LPI), lyso-phosphatidylglycerol (LPG), and lyso-phosphatidate (LPA) showed no significant change of relative abundance after maturation (**Figures 3** and **4D**). All values of lipid class mol% are further shown in **Table 1**. Thus, we conclude that moDCs exhibit an overall shift of membrane and storage lipids upon maturation.

**Table 1:** pmol values of lipid classes of immature and mature moDCs.

Class	Immature	Mature	Immature_sd	Mature_sd	Aov_pval
CE	0.122	0.082	0.097	0.015	0.507
Cer	0.151	0.212	0.068	0.013	0.012
CL	0.593	0.507	0.121	0.139	0.157
DAG	0.398	0.552	0.111	0.286	0.130
HexCer	0.050	0.138	0.019	0.051	6.76E-05
LPA	0.046	0.007	0	0	
LPC	0.013	0.018	0.006	0.007	0.186
LPE	0.008	0.009	0.003	0.003	0.411
LPE O-	0.006	0.008	0.003	0.002	0.073
LPG	0.011	0.009	0.002	0.002	0.209
LPI	0.036	0.038	0.013	0.009	0.793
PA	0.054	0.086	0.045	0.062	0.297
PC	44.614	46.612	1.529	2.005	0.022
PC O-	7.169	6.202	1.155	0.878	0.049
PE	9.381	8.075	0.403	0.480	3.47E-06
PE O-	13.324	13.577	0.631	0.635	0.384
PG	0.556	0.442	0.111	0.097	0.025
PI	7.718	7.025	0.684	0.472	0.017
PS	6.821	6.835	0.513	0.843	0.965
SM	8.255	7.391	0.454	0.424	3.49E-04
TAG	0.783	2.253	0.499	1.125	0.001

### Maturation Leads to a Reduction of Plasma Membrane Lipid Packing in Mature moDCs

**Figure 5:**
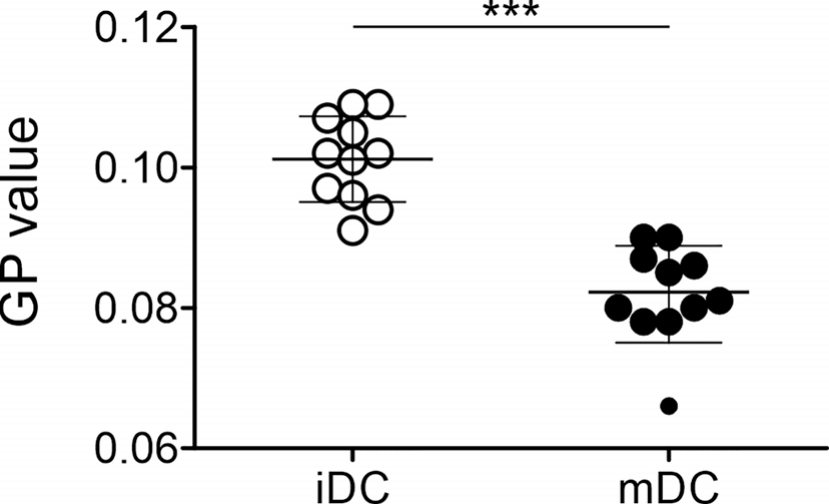
Differential lipid order of plasma membrane of immature and mature moDCs. Lipid packing imaging of immature and mature moDCs was performed. Therefore, cells were spiked with a final concentration of 0.4 μM with Di-4-ANEPPDHQ. The spectral imaging was performed using a Zeiss LSM780 confocal microscope equipped with a 32-channel GaAsP detector array. Fluorescence excitation of Di-4-ANEPPDHQ was set to 488 nm and the lambda detection range was set between 500 and 700 nm. The values of the 32 channels were analyzed within the ImageJ plug-in “Stacks-T functions-Intensity vs. Time Monitor”. To calculate generalized polarization (GP) values, the wavelengths λ = 650 nm as maximum wavelength in disordered membraned (red shifted) and λ = 560 nm as maximum wavelength in the gel phase (blue shifted) were used as described previously (77, 78).
$\text{GP}\;=\frac{I_{560}-I_{650}}{I_{560}+I_{650}}$Statistical significances were calculated using paired t-test (n ≥ 10). Not significant (n.s.) p > 0.05, *p < 0.05, **p ≤ 0.01, ***p ≤ 0.001.

As mature moDCs were stiffer than immature cells, we aimed to understand whether the plasma membrane of moDCs might still provide interaction flexibility. To address this question, we performed lipid packing imaging. To this end, immature and mature moDCs (day 7) were stained with a membrane-embedded polarity-sensitive dye Di-4-ANEPPDHQ, whose emission spectrum is dependent on the molecular order of the immediate membrane environment (79). Polarity in biomembranes generally represents the hydration level of the bilayer and therefore the order of membranes (87, 88). Spectral imaging was performed utilizing a Zeiss LSM780 confocal microscope equipped with a 32-channel GaAsP detector array and the relative order of lipids (lipid packing) in the membrane of moDCs was analyzed for their general polarization (GP) value (77). We found that mature moDCs (0.082 ± 0.008) displayed a significantly lower GP value than immature moDCs (0.101 ± 0.006) (**Figure 5**). Thus, our data suggest that mature moDCs harbor a higher fluidity and therefore a higher polarity of the plasma membrane based on the content of water.

Overall, our lipidomics screening together with measurements of the mechanical properties revealed that the higher stiffness of mature moDCs is accompanied by an increase in membrane fluidity, thus potentially providing the plasticity required for immunological processes including the formation of an immunological synapse in mature antigen-presenting DCs.

## Discussion

As the maturation of DCs is a highly complex process that involves various changes at the global cellular level (5, 89), we here aimed to investigate whether the DC maturation process also influences the cell mechanics of DCs as well as the global lipid composition and consequently, membrane properties. We hypothesized that the mechanical properties of DCs might change upon maturation in order to detach form peripheral tissues, to migrate into lymph nodes, and to withstand mechanical and shear stress during this process. We further postulated that changes in lipid content in plasma membrane or general cellular lipid content, might contribute to the adaption of the cells associated with maturation processes. We here demonstrate by real-time deformability cytometry analyses that the cellular stiffness of moDCs is increased after the process of maturation. The observed changes upon moDC maturation in cellular mechanics were accompanied by a remodeling of the total lipidome. Beside these changes, the LDL content of the donor’s serum was associated with a strong effect on the total lipid composition of moDCs. Moreover, our data display higher plasma membrane fluidity that is related to the increased phosphatidylcholine (PC) and decreased sphingomyelin (SM) lipid content upon maturation.

As it was suggested that the cortex is connected to the actin cytoskeleton beneath the plasma membrane and plays a central role in cellular shape control (90), we first performed mechanical phenotyping experiments to investigate into the overall cell mechanics of moDCs during maturation. We used real-time deformability cytometry since this method is able to quantitatively analyze cell material properties such as cell stiffness and was found to be sensitive to cytoskeletal alterations (80). As it is still unknown where and how these maturation processes takes place, we investigated immature and mature (24h stimulation with maturation cocktail) moDCs that represent two definite time points of the maturation status of cells. Here, we identified that the overall cell mechanics of moDCs has changed. These data are suggesting that mature moDCs displayed a higher overall stiffness than immature moDCs (**Figure 1**). Our findings are consistent with recent data received from applying atomic force microscopy measurements on murine bone marrow-derived dendritic cells and primary DCs from murine spleens (53). However, although atomic force microscopy is a sophisticated method to characterize mechanical properties of cells, an advantage of real-time deformability cytometry is the high-throughput. Furthermore, atomic force microscopy requires the attachment of cells to a surface, potentially influencing their mechanical properties. Thus, deformability cytometry provides a more cell autonomous measurement as the cells are fully suspended in solution. From our experiments, we speculate that the increased stiffness of mature moDCs might help the cells to travel through different constrictions in lymphatic vessels in a fast manner on their way to the lymph nodes and to increase their robustness with respect to the shear stress during migration processes. This would be consistent with interesting data of Barbier et al., which indicate that mature murine bone marrow-derived DCs are able to squeeze through small 3D microchannels at high speed (91). Inversely, our data suggest that immature moDCs appeared to be less stiff, suggesting that they need this higher flexibility of their cytoskeleton to sense, embrace fragments, and uptake antigens in peripheral tissues. This is in accordance to findings by Heuzé and Vargas, in which micropinocytosis or endocytosis have been suggested to be dependent on the membrane-cytoskeletal interface including alternating phases of low and high motility in the immature state (92). Although the cell size differences were not statistically significant, the smaller size of mature moDCs might have an effect on the overall cell mechanics such as cellular robustness and migration (**Figure 1E**).

As important antigen-presenting cells, the main function of DCs is the activation of T cells. Previous results indicated that the induction of T cell activation and profound proliferation needs a prolonged contact of the T cell with an antigen-presenting cell (93, 94). This process is supported by the formation of a so-called immunological synapse (95, 96). Indeed, it was suggested that the cellular mechanics also play an important role in the formation of a synapse. This was in line with the fact that the cytoskeleton of DCs was found to be rearranged after stimulation leading to the relocation of filamentous actin to the site of the immunological synapse (54, 97–99). Blumenthal et al. further showed that the increase in cortical stiffness of mature bone marrow-derived DCs is also due to the actin cytoskeleton modulation (53). As we agreed that a higher stiffness of maturing moDCs is useful to increase resistance to shear stress during the process of DC migration and by providing physical resistance to the pushing and pulling forces exerted by the interacting T cell, we wondered how the higher stiffness of mature moDCs matches the formation of an immunological synapse and the interaction with T cells, a cellular process that needs a high membrane flexibility (100). Thus, we measured the order of the plasma membrane using lipid packing imaging. Complementing the cell mechanics studies, where mature moDCs were stiffer than immature cells, our data revealed that mature moDCs exhibited a lower GP value than immature cells, suggesting a more disordered membrane (**Figure 5**). In general, mature DCs have to be stiff in order to fully activate T cells, controlled by the cortical cytoskeleton (53); however, the order of the plasma membrane is independent from cortical stiffness and might be more fluid with a higher diffusion capacity to rearrange molecules that are important for the initiation of the immunological synapse. This was also in accordance with data from Ayee et al. who demonstrated that an increase in the rigidity and lipid packing of the membrane does not translate into an increase in the overall stiffness of the cellular cortex. On the contrary, it appears that there is an inverse relationship between lipid order of the membrane bilayer and the global stiffness that is dominated by the cortical cytoskeleton (101). The lipid packing of moDCs was a highly interesting finding as the membrane fluidity seems to be uncoupled from the overall stiffness of the cell. In agreement, Blanchard et al. suggested that the two properties of cell deformability and membrane fluidity might play complementary roles in immune cells (102).

Besides cytoskeleton and molecular changes, such as upregulation of costimulatory molecules, lipids might also influence the DC’s function during maturation. Lipids play a fundamental role in cell homeostasis and regulation and have diverse functions. They form biological membranes, where they are directly involved in intracellular trafficking, membrane compartmentalization, and the interaction or function of membrane proteins. Further, lipids can act as signaling components themselves (66, 85). Here, we performed high-throughput shotgun lipidomics to investigate the lipidome of immature and mature moDCs of ten different healthy donors. To our knowledge, this is the first study investigating the lipidome of human moDCs. In total, 830 lipids were analyzed. Noteworthy, the total lipid amount did not change during maturation (**Figure 2B**). Since we could not detect differences in the quantity of lipids, we were further investigating the lipid quality. By comparing all lipid species in the hierarchical cluster analysis, we found that mature moDCs clustered away from immature moDCs (**Figure 2C**). From these data we concluded that the maturation status of the cells had the highest impact among all factors (comprising gender and serum lipid content) on the global lipid composition of moDCs. In addition, subclusters within the maturation dependent clusters indicated a second component influencing the global moDC lipid composition.

To corroborate the findings from hierarchical clustering and to reduce the complexity of lipidomics data, we further performed PCA. Our results clearly revealed that the maturation status separated the donors into groups of immature and mature moDCs (**Figures 2D–G**). Therefore, we analyzed the clinical parameters of the beforehand obtained serum samples of the same donors (which was taken 7 days before the moDC lipidomics analysis). Although not unexpected but still surprising, we found that the serum lipids were fluctuating between individual donors (**Figure 2H**) most likely owed to time points of thrombocyte apheresis and eating habits, life style, and genetic predispositions (103, 104). Furthermore, when we overlaid our metadata with the PCA data, we found that independent of the moDC maturation status, serum LDL-C (and to a lower extent also cholesterol levels) clustered the donors into two different groups: those with high LDL-C and those with low LDL-C serum levels. This was a very intriguing result, since the lipid composition of low and high LDL-C donors is conserved after seven days in cell culture that are needed for the generation of moDCs. This might indicate an imprinting effect of the serum lipid levels on the immune cells including moDCs. As cholesterol homeostasis has a pivotal function in regulating immune cells and since an accumulation of cholesterol in the cell membrane of moDCs has been suggested to enhance MHC II-dependent antigen presentation and CD4^+^ T-cell activation (105), further analyses of high and low LDL-C donors might help to elucidate whether changed lipid serum levels also have an impact DC-mediated immune responses.

When we investigated all lipid classes in more detail (**Figures 3**, **4**), we found that the membrane sphingolipid ceramide (Cer) showed a significantly higher lipid content in mature moDCs. Ceramide is formed under all conditions of cellular stress and plays a role in signal transduction as lipid second messenger (85, 106). Moreover, ceramide might also be involved in the inhibition of antigen uptake upon DC maturation (107). Strikingly, ceramide-enriched membranes are ideal to sort proteins and to provide an environment for spatial organization of receptors. In fact, ceramide-dependent receptor clustering was already shown for CD40 and CD40 ligand pairs in moDCs and interestingly, these molecules are involved in the formation of the immunological synapse (108–110). Altogether, this supports the notion that the increase of ceramide upon maturation might help moDCs to form an immunological synapse with T cells with regard to stability and a prolonged contact of DCs with T cells (109, 111).

The increase of ceramide was accompanied by a decrease of sphingomyelin (SM) in mature moDCs due to their closely linked metabolism, as ceramide constitutes the backbone for sphingomyelin. Sphingomyelin is found in membranes and associates with proteins and cholesterol in membrane rafts, which display more ordered membrane parts (62, 112). This behavior is also in accordance with our lipid packing analysis, since a higher sphingomyelin level in immature moDCs is associated with a stiffer membrane (**Figure 5**). In addition, sphingomyelin seems to interact with caveolae that form compartments for endo- and exocytosis in the plasma membrane (113, 114). Thus, sphingomyelin might also play a role for antigen uptake, which explains its higher levels in immature moDCs. Interesting lipidomics data from Santinha et al. suggest a correlation between sphingomyelin and ceramide that appears upon differentiation of DCs and that influences the immunomodulatory properties of DCs including T cell activation (115).

The most prominent lipid in the cell and especially in membranes is phosphatidylcholine (PC). The relative abundance was increasing during maturation of moDCs. In particular, phosphatidylcholine synthesis is required to stabilize the surface of so-called lipid droplets (or lipid bodies) in tissues, where triacylglycerols (TAG) are stored (85). Accordingly, triacylglycerol levels were also significantly increased following maturation of moDCs. This was an interesting finding as triacylglycerol represents an energy storage and might thus play an important role in the metabolism of DCs. Our finding is consistent with previously shown data that triacylglycerol and lipid droplets are accumulating after activation of myeloid cells, indicating an influence on the metabolism of DCs after TLR stimulation (116–118). Overall, lipids accomplish complex functions in DCs, depending on the maturation state of the cell.

The observed lipid class shift of immature to fully mature moDCs might depend on several processes. One of these processes could be the remodeling of cellular membranes, as it is well known that the maturation process induces the transport and fusion of MHC II containing vesicles to the plasma membrane (119). Further, processes such as metabolic changes occur during DC maturation, in which a switch from oxidative phosphorylation to glycolysis might influence the lipid biosynthesis (120, 121). Thereby, one could speculate that a *de novo* synthesis of fatty acids might induce a shift of the lipid composition during maturation of moDCs. Future experiments will be necessary to evaluate the exact mechanism how the observed shift of the lipid composition is mediated. This might be possible by cell fractionations to isolate membrane-containing compartments of immature and mature moDCs followed by lipidomics.

Although *ex vivo* generated moDCs are widely used for immunotherapeutic approaches (17, 23, 25, 34, 37) – their efficacy in including desired anti-tumor responses still needs to be improved (15, 23, 38). For example, the efficiency of tumor antigen loaded matured moDCs to migrate and settle into lymph nodes is not fully accomplished, since the majority of moDCs does not reach the lymph node (122). In addition, also the immunosuppressive microenvironment of tumors might interfere with the efficacy of the moDC therapy, especially in regard to their capability to induce or to reactivate tumor T cell responses (123). The aspect, that cholesterol impacts on the function of our immune system was recently described for NK cells (124). Interestingly, as serum LDL-C levels seem to influence the lipid composition, irrespective from the maturation status of moDCs, further studies will be necessary to evaluate the initiation of immune responses from moDCs of high and low serum LDL-C donors that might impact T cell and DC activation, or DC migration. For future immunotherapeutic approaches, the cellular and/or serum lipid content of patients could serve as a biomarker to identify patients who are suitable for therapy using autologous cells transfer. In addition, real-time deformability cytometry might help to evaluate the moDC quality before application in cancer patients.

*In vitro* generated moDCs are not fully comparable to primary steady-state DC subsets present in the body (125); instead, they rather resemble the cells that appear during an inflammatory response *in vivo* (21, 126, 127). Moreover, human inflammatory DCs display conserved gene signatures with moDCs and present a distinct subset of DCs (21). Thus, future experiments need to be performed comparing moDCs and primary DC subsets for lipid content and cell mechanical properties. Primary DCs or *in vitro* expanded primary DCs (naturally occurring DCs) constitute the next generation of DC therapy. However, it is still unclear which particular DC subset might be used for different immunotherapeutic approaches (128). Thus, moDCs as well as these next generation DC vaccines might serve as combinatorial therapeutics for immune checkpoint inhibitors (128–131).

In conclusion, our study provides the first major observations for maturation dependent changes of moDCs using cutting-edge techniques such as real-time deformability cytometry as well as shotgun lipidomics analysis. Future experiments are needed to fully link the cell function, mediated by the cytoskeleton, to the increased cell stiffness within mature moDCs. Furthermore, the changes in lipid composition and in membrane properties of moDCs need follow-up studies to understand how they might influence the immune response initiated by moDCs. Additionally, this study provides the rationale to consider the easy accessible parameters of cellular deformation and serum lipid content as potential future biomarkers allowing for suitability prediction of a given patient for autologous cell transfer therapy. Our results are just the beginning in understanding the bigger picture of moDC maturation where each of the changes of cellular properties might contribute to multiple functions.

## Data Availability Statement

The raw data supporting the conclusions of this article will be made available by the authors, without undue reservation. Lipidomics raw data are included in the **Supplementary Material**.

## Ethics Statement

The studies involving human participants were reviewed and approved by Ethikkommission der Friedrich-Alexander-Universität Erlangen-Nürnberg. The patients/participants provided their written informed consent to participate in this study.

## Author Contributions

JL performed the experiments with participation by LH, GH, MKr, MKu, and ES. NA and CL analyzed lipidomics data. LH, LA, and CL contributed to the review of the manuscript. LA, CL, and DD contributed to the data analysis and interpretation as well as discussions. JL and DD designed the study, JL, LA, and DD wrote the manuscript, and CL, LH, GH, MKr, MKu, ES, A-SS, VZ, RB, IL, MD, CE, and JG critically revised the manuscript. All authors contributed to the article and approved the submitted version.

## Funding

This work was partly supported by grants from the German Research Foundation [Deutsche Forschungsgemeinschaft (DFG)] to DD (CRC1181-TPA7, DU548/5-1), to DD, RB, and A-SS (RTG1962); the Emerging Fields Initiative BIG-THERA of the Friedrich-Alexander University Erlangen-Nürnberg to DD and A-SS; Erlanger Leistungsbezogene Anschubfinanzierung und Nachwuchsförderung (ELAN) (DE-17-09-15-1-Heger) to LH; Interdisziplinäres Zentrum für Klinische Forschung (IZKF) (IZKF-A80) to DD; Wellcome and Kennedy Trust for Rheumatology Research (PRF 100262Z/12/Z) to MD. ES is supported by SciLifeLab fellow program. CE acknowledges imaging support by the Wolfson Imaging Centre – Oxford, and funding by the Wolfson Foundation, the EPA Cephalosporin Fund, MRC (Grant No. MC_UU_12010/unit programs G0902418 and MC_UU_12025), the Wellcome Trust (Grant No. 104924/14/Z/14 and Strategic Award 091911 (Micron)), MRC/BBSRC/EPSRC (Grant No. MR/K01577X/1), the John Fell Fund, state of Thuringia (Thüringer Aufbaubank (TAB)), the Deutsche Forschungsgemeinschaft (Research unit 1905, Jena Excellence Cluster “Balance of the Microverse” and Collaborative Research Center 1278) and the Jena Center of Soft Matter.

## Conflict of Interest

The authors declare that the research was conducted in the absence of any commercial or financial relationships that could be construed as a potential conflict of interest.
